# Do Anti-Egalitarians Report Increased Support for People with Language Difficulties when Exposed to Gender-Fair Language?

**DOI:** 10.5334/pb.1342

**Published:** 2025-05-22

**Authors:** Pascaline Van Oost, Kenzo Nera, Vincent Yzerbyt

**Affiliations:** 1 Institute for Research in the Psychological Sciences, Universitécatholique de Louvain, Place Cardinal Mercier 10 box L3.05.01, 1348 Louvain-la-Neuve, Belgium; 2 Faculty of Psychological Sciences and Education, Universitélibre de Bruxelles, Avenue F.D. Roosevelt 50, 1050 Bruxelles, Belgium

**Keywords:** gender-fair, language, social dominance, justification, sexism

## Abstract

In many countries, the use of gender-fair language is heavily debated. In France, some opponents to gender-fair language have argued that it hinders language comprehension for people who have difficulties with language (PDLs). This argument was notably promoted by (far) right-wing personalities and newspapers. The justification-suppression model of prejudice and the concept of ideology malleability suggest that such a defence of PDLs may be a strategy to oppose gender-fair language and promote the status quo. We hypothesized that threatening participants with gender-fair language would lead high-SDO individuals to report greater concern for PDLs. In two experimental studies (n_total_ = 1117, France), we did not find support for our prediction. Overall, SDO was negatively correlated with support for PDLs, whereas participants supporting gender-fair language were also more concerned with PDLs. This suggests that contrary to what some conservative commentators have claimed, gender-fair language supporters do not overlook the question of language accessibility, as opposed to anti-egalitarians. To our knowledge, this is the first research to bridge literature on the justification of prejudice and gender-fair language.

## Introduction

In the French-speaking world, the use of gender-fair language (‘*langage inclusif*’) is heavily debated. Gender-fair language, often referred to as ‘non-sexist language’ or ‘gender-inclusive language’, encompasses linguistic practices aimed at equally representing women, men, and sometimes other gender categories. Public attention in France was drawn to gender-fair language in 2017 when a French publisher released a textbook for 7-year-old pupils using such language. This issue gained public and political attention after coverage by a right-wing media outlet. At the time, the minister of education expressed a concern that gender-fair language ‘added a complexity that is not needed’ and that it would in fact hurt gender equality (BFM, 2017). Two months later, the prime minister issued a circular limiting its use in administrative and teaching settings (Circulaire du 21 novembre 2017 relative aux règles de féminisation et de rédaction des textes publiés au Journal officiel de la République française, 2017). Today, gender-fair language use is still a subject of debate in France, as French deputies examined a ban on the gender-fair language in February 2024. Interestingly, while gender-fair language encompasses several techniques such as the feminization of masculine forms of job names (e.g., ‘*pompière*’ for ‘firewoman’), the combination of masculine and feminine forms (e.g., ‘*étudiantes et étudiants*’), the use of gender-unmarked forms (e.g., ‘*corps étudiant*’), etc., the ‘*point médian*’ (collapsing the masculine and feminine forms, e.g., ‘*étudiant·e·s*’) received most of media attention, such that gender-fair language was arguably often reduced to this specific technique (as a case in point, the circular issued by the former education minister purportedly banning ‘*écriture inclusive*’ actually targeted this technique only; Abbou et al., 2018).

Several motives may underlie opposition to gender-fair language. A first, perhaps obvious one, is a desire to maintain hierarchy. Indeed, the main role of gender-fair language is to tackle, or even suppress, gender inequality within the language. Individuals who prefer hierarchy (i.e., social-dominance oriented individuals) will likely tend to dislike the hierarchy-attenuating goal of gender-fair language. Probably because of its non-normative, antiegalitarian nature, this argument is hardly present in public discourse. Rather, the main arguments brought up by opponents to the use of gender-fair language are threefold. First, gender-fair language is said to be a threat to the French language, and to the continuity of the French culture. Anne-Laure Blin, a right-wing member of Parliament who proposed a bill to forbid gender-fair language, declared: ‘I alerted my 576 colleagues and asked them to co-sign this text regardless of their political allegiance. The issue is not political but national (…) in the interests of the French people and the preservation of our language’ (our translation, Ouest France, 2022). Second, gender-fair language is said to be inefficient in reducing sexism. However, research in psycholinguistics has shown, in general, positive effects of feminization and other forms of gender-fair language (see Gygax et al., 2013 for a review on the interpretation of gender marking and the effects of gender-fair language, but see Formanowicz et al., 2013 for unwanted side effects of language feminization in specific contexts). Third – and this argument is the focus of this research – gender-fair language allegedly complexifies the language and hinders language learning, particularly for people encountering difficulties with language (e.g., dyslexia). In 2021, the *Acad*é*mie Fran*ç*aise* director and secretary wrote ‘Gender-fair language (…) has the effect of creating a second language, the complexity of which penalises people with cognitive disabilities such as dyslexia, dysphasia or apraxia. An apparent petition for justice has the concrete effect of exacerbating inequalities’ (our translation, Carrère d’Encausse & Lambron, 2021). Perhaps ironically, this argument was notably promoted by (far) right-wing personalities and newspapers. The French newspaper *Valeurs Actuelles*, a far right-wing publication, headlined: ‘Gender-fair language: an illegible and penalising form of writing for many disabled people’ (our translation, Valeurs Actuelles, 2020). During a senate debate about gender-fair language, Stéphane Ravier, a politician from the Rassemblement National, a far-right French party, argued that ‘Gender-fair language is nothing other than exclusionary writing, which puts the French language at risk. The visually impaired, dyslexics and foreign students will be the victims of this destruction’ (our translation, Public Sénat, 2021). During this session, Thomas Dossus, a left-wing member of parliament, alluding to a hypocritical defence of these disadvantaged groups, declared: ‘This sudden passion for children with disabilities magically disappears when it comes to raising the salaries of carers for pupils with disabilities’ (our translation, Public Sénat, 2021).

This concern for people with a language disability in the context of gender-fair language can be viewed as surprising, considering that right-wing political views usually correlate with reduced concern for disadvantaged groups (Jost et al., 2009). One may therefore wonder if the concern for people with language difficulties is not contextually mobilized by antiegalitarians to justify an opposition to gender-fair language, and the associated political agenda (i.e., the promotion of equality). In other words, one may wonder whether the argument of language difficulties is brought up by antiegalitarian wishing to maintain hierarchy. This possibility is captured in the model of justification-suppression model of prejudice (Crandall & Eshleman, 2003) and in the concept of malleable ideologies (Knowles et al., 2009). The first suggests that individuals are likely to turn to justifications to express their prejudice. Knowles and colleagues found that ideologies can serve this purpose. Specifically, they found that antiegalitarian individuals sometimes manifest an opportunistic endorsement of egalitarian values (e.g., colorblindness, diversity, *la*ï*cit*é, Knowles et al., 2009; Roebroeck & Guimond, 2018; Unzueta et al., 2012) when facing threat to the status quo. Building on this concept, we tested the hypothesis that antiegalitarians may express an opportunistic defence of PDLs, that is, whether their endorsement emerges in situations where the issue of gender-fair language is made salient.

### Gender-fair language acceptance

Acceptance or resistance towards gender-fair language practices were investigated in several languages and countries during the last decades, highlighting both individual and contextual factors. Gender-fair language acceptance is associated with lower levels of sexism in American, British, Canadian, Swiss or Swedish samples (Douglas et al., 2019; Gustafsson Sendén et al., 2015; Jacobson & Insko, 1985; Martyna, 1978; Matheson & Kristiansen, 1987; Parks & Roberton, 2004; Sarrasin et al., 2012; Swim et al., 2004). In a similar vein, it was associated with lower levels of social dominance orientation and gender-specific system justification among British undergraduates (Douglas & Sutton, 2014). Somewhat surprisingly, results regarding the effect of participants’ gender are mixed, with some evidence suggesting that men exhibit lower level of acceptance of gender-fair language (Douglas & Sutton, 2014; Gustafsson Sendén et al., 2015; Jacobson & Insko, 1985; Matheson & Kristiansen, 1987; Parks & Roberton, 2004), and others finding no differences (e.g. Sczesny et al., 2016; Vervecken & Hannover, 2012). As for age, some studies show that it is negatively related with acceptance of gender-fair language (e.g., Gustafsson Sendén et al., 2015). However, Parks and Roberton (1998) found that people 23 years of age and older were significantly *more* supportive of gender-fair language compared to younger participants.

Besides individual characteristics, a few studies have highlighted the importance of contextual factors in the acceptance of gender-fair language. For example, comparing Austria and Poland, Formanowicz and colleagues (2015) found that in Poland, where gender-fair language is rare, social initiatives (i.e., quotas) were rated less favorably when gender-fair (i.e., feminine) forms were used instead of traditional masculine forms. Conversely, in a context where gender-fair language is well established (i.e., Austria), similar initiatives were evaluated more positively when women were presented with gender-fair (feminine) forms. On a somewhat different note, Gustafsson Sendén and colleagues (2015) found that attitudes towards a newly-introduced Swedish neutral pronoun ‘hen’ improved significantly over time. In fact, in this study, time was the most important predictor of attitude improvement (even after controlling for factors such as gender, sexism, political orientation).

In sum, results suggest that attitudes towards gender-fair language evolve with time, but that individuals harboring antiegalitarian views will tend to reject it to a greater extent.

### Ideologies as justifications

A well-established finding in the work on stereotyping and discrimination is that individuals are motivated to view themselves as unbiased and devoid of prejudice (Monteith et al., 2010), and are more likely to express prejudice when they can provide a socially acceptable justification for it (Crandall & Eshleman, 2003). Hence, individuals with high social dominance levels or high prejudice levels may strategically mobilize socially accepted ideologies to justify their antiegalitarian views (Crandall & Eshleman, 2003; Knowles et al., 2009). Coining the term ‘malleable ideologies’, Knowles and colleagues (2009) have shown in a series of studies that high-SDO whites exposed to intergroup threat (e.g., reduced economic opportunities for white people at the benefit of Black people) shift their conception of colour-blindness. Specifically, by endorsing a procedural form of colour-blindness whereby strict equal treatment is emphasized, antiegalitarian can legitimize a preference for the racial status quo and dismiss affirmative action policies. In a similar vein, White and Crandall (2017) found that prejudiced participants adhere to the ‘free speech’ principle when exposed to anti-black hate speech, but less so when the hate speech targets the police, showing some form of arbitrary endorsement of this important value. Closer to our context of interest, Roebroeck and Guimond (2018) examined the support for *la*ï*cit*é in France, a once egalitarian principle that was recently reinterpreted as proscribing the wearing of religious signs (e.g., the hijab) in a growing number of settings. These authors found that high-SDO participants report more attachment to *la*ï*cit*é when faced with symbolic threat – a ‘symptom of intolerance’ (Roebroeck & Guimond, 2018, p. 13). Along parallel lines, Van Oost et al. (2023) examined how gender equality, a principle often said to clash with Islamic practices, can be ironically embraced by less egalitarian Belgians in their effort to justify anti-Islam stances. Using a discourse analysis, they found indeed that antiegalitarian participants recruit the principle of gender equality when elaborating on their negative attitudes towards Islam.

In sum, these results suggest that antiegalitarians can recruit egalitarian ideologies or principles in their effort to justify their prejudiced stances. Building on this research, the present paper aims to examine whether antiegalitarian individuals will recruit the inclusion for PDLs when threatened by gender-fair language, a practice that aims to challenge the gender status quo embedded in language. We tested this hypothesis in two experimental studies.^1^ The research adhered to all ethical concerns outlined in the Helsinki Declaration.

## Study 1

In Study 1, we hoped to induce a sense of threat from gender-fair language among high SDO participants. Participants in the experimental condition read a vignette about gender-fair language, written in gender-fair language, whereas participants in the control condition read a vignette about a neutral subject. Participants then indicated their level of support for the inclusion of PDLs, as well as another, more ecological dependent variable, namely support for an individual struggling with language voicing concerns about the current usage of French. We expected that participants exposed to the ‘gender-fair language’ vignette would think about the issues that gender-fair language can cause for a PDL. Experimental participants should likely interpret the person’s complaint as specifically related to gender-fair language. Conversely, control participants should perceive the complaint in a more general context. This approach allowed us to determine whether participants’ support for the person with language difficulties is contingent on the involvement of gender-fair language or if their support remains consistent regardless of the issue at hand. Preregistration, materials, data files and analyses scripts are available on the Open Science Framework: https://osf.io/rw7cg/?view_only=fdd70967d6ac4211b29784cc13522c82.

### Hypotheses

We hypothesized that SDO would be negatively related to the inclusion of PDLs (H1) and to the support of the PDL person complaining (H1b). We predicted that favorability to gender-fair language would be positively related to the inclusion of PDLs (H2) and the support for the PDLs person complaining (H2b). Furthermore, we expected an interaction between SDO and condition to predict the inclusion of PDLs (H3) and between condition and the support for the PDL person (H3b). Specifically, we expected that high-SDO participants would have a more positive attitude towards the inclusion of PDLs and will support more the person who complains about French when they have been exposed to the issue of gender-fair language (i.e., experimental condition) compared to a condition where they not have been exposed to the issue of gender-fair language (i.e., control condition). We expected that low-SDO participants would have an equally positive attitude towards inclusion of PDLs in both conditions.

### Methods

#### Participants

We sought to achieve 80% power to detect a small minimum effect size (*r* = .10) (Funder & Ozer, 2019). To improve data collection efficiency while controlling type 1 error risk, we made use of sequential analyses (Lakens, 2014). Thus, we planned to collect a maximum of 800, with an interim analysis planned halfway through sample completion (n = 400) to have an equal number of observations between each look (Lakens, 2014).

We interrupted data collection at the intermediate sample size as was preregistered because we did not observe the expected significant effects, even when doubling the sample size by duplicating observations. As recommended in the Pocock boundary method, the interim analyses were conducted with the adjusted p-value threshold of 0.0294.

We recruited participants using Foule Factory (*n* = 396), a French crowd-sourcing platform. After excluding participants who failed the attention checks, our sample comprised 389 participants (187 women, 185 men, 2 who identified to another gender, *M_age_* = 43.10, *SD_age_* = 12.7). In terms of education level, 30.9% had no diploma, a primary or secondary school diploma, 25.1% had completed one to three years of higher education, 30.2% had completed 4 years of higher education or more.

#### Procedure

After giving their informed consent, participants completed demographics questions (gender, age, political orientation, level of education) and the social dominance orientation scale. Then, participants were randomly assigned to one of two conditions. Experimental participants read a vignette about the use of gender-fair language. Control participants read a vignette about albatross birds. The material can be found in the appendices. Following the experimental manipulation, participants were shown the screenshot of a bogus Facebook status written by a man named Pierre, stating ‘the current use of the French language is really not accessible for people who struggle with French like me’. They were asked to indicate their support for this person. Then, they filled the scale measuring the inclusion of persons who have difficulties with language, the manipulation check, and finally, a gender-fair language attitude scale. They were then thanked and debriefed.

#### Measures

Unless mentioned otherwise, participants responded to the items on a 7-point Likert scale ranging from completely disagree (1) to completely agree (7), with 4 indicating ‘neither agree nor disagree’.

**Social dominance orientation (**α **= .91)**. Participants responded to SDO scale adapted to French by Duarte et al. (2004). We used the 10-item version of this scale from Troian et al. (2018). An example item was ‘It’s probably a good thing that some groups are on top and others are on the bottom’.

**Support to a PDL person complaining about the current usage of French**. Participants indicated their level of support to a man complaining about the current used of French in a fake Facebook status. Specifically, participants were shown a fake Facebook status of a French man stating: ‘the current use of the French language is really not accessible for people who struggle with French like me’. Then, they were asked ‘to what extent do you support this man in what he says?’. They indicated their support level with a scale ranging from 1 (‘not at all’) to 7 (‘completely’).

**Inclusion of persons who have difficulties with language (PDLs**, *α* **= .90)**. Participants completed a 9-item scale measuring their desire to better include people encountering difficulties with language in society. The question read: ‘In the following statements, we refer to people with language difficulties. We are referring here to people with dyslexia, mental disabilities or low literacy’. An example item was ‘Society does far too little to include people with language difficulties’.

**Manipulation check**. To check whether participants in the experimental condition reported higher concern about the use of gender-fair language, we asked ‘to what extent are you currently worried with the following things?’ on a 7-point Likert-type scale: ‘the space that the English language is taking and the ‘*anglicisation*’ [i.e., the growing use of English words in French]’; ‘the use of gender-fair language’; ‘the simplification of French with the new writing’; ‘the overall mastery of the French language among younger generations’. We hypothesize that levels of worries on the item ‘the use of gender-fair language’ would be higher in the experimental condition.

**Gender-fair language attitudes (*α* = .81)**. Participants responded to three items taken from the gender-fair language attitude scale from the ’Inventory of Attitudes Towards Sexist/non-Sexist Language-General’ (Parks & Roberton, 2000) which we adapted to the French-speaking context. (‘Worrying about the sexist nature of the French language is really a futile concern’, ‘the [*point médian*] (for example, ‘cher-e-s ami-e-s’) is a viable way of making French less sexist’ and ‘When referring to a mixed group, it is acceptable to use the [*point médian*] (for example: [‘*les musicien·ne·s de l’orchestre*’])’). We selected these items based on the psychometric properties they showed in the Supplemental study conducted before Study 1.

### Results

Table 1 presents a correlation matrix. Attitudes towards gender-fair language were negatively associated with SDO, *r* = –.17, *p* < .001, and positively associated with inclusion of PDLs, *r* = .27, *p* < .001. Positive attitudes towards gender-fair language were also associated with support for the person complaining about French being inaccessible, *r* = .19, *p* < .001. Positive attitudes towards gender-fair language and inclusion of PDLs showed a positive association, *r* = .27, *p* < .001. In contrast, SDO was negatively associated with inclusion of PDLs, *r* = –.36, *p* < .001, but was not associated with support for the PDL complaining, *r* = –.08, *p* = .14.

**Table 1:** Means, standard deviations, and correlations with confidence intervals (Study 1).


VARIABLE	*M*	*SD*	1	2	3	4	5	6	7

1. Gender (–0.5 = man, +0.5 = woman)	*na*	*na*							

2. Age	42.93	12.56	–.01						

			[–.11, .09]						

3. Education level	6.97	2.47	–.13*	–.14**					

			[–.23, –.03]	[–.24, –.04]					

4. Political orientation	3.91	1.49	–.05	.06	–.11*				

			[–.14, .05]	[–.04, .16]	[–.20, –.01]				

5. SDO	2.31	1.10	–.11*	–.06	–.07	.43***			

			[–.21, –.01]	[–.15, .04]	[–.17, .03]	[.35, .51]			

6. Support for the PDL person	3.51	1.80	–.00	.05	–.02	–.17**	–.08		

			[–.10, .10]	[–.05, .15]	[–.12, .08]	[–.26, –.07]	[–.17, .02]		

7. Inclusion of PDLs	5.21	1.17	.09	.01	–.13*	–.23***	–.36***	.35***	

			[–.01, .19]	[–.09, .11]	[–.22, –.03]	[–.33, –.14]	[–.44, –.27]	[.26, .44]	

8. Attitudes towards gender-fair language	3.43	1.73	.11*	–.14**	–.11*	–.40***	–.17***	.19***	.27***

			[.01, .20]	[–.24, –.04]	[–.21, –.01]	[–.48, –.32]	[–.27, –.07]	[.09, .28]	[.17, .36]


*Note. M* and *SD* are used to represent mean and standard deviation, respectively. Values in square brackets indicate the 95% confidence interval for each correlation. * indicates *p* < .05. ** indicates *p* < .01. *** indicates *p* < .001.

**Figure 1:**
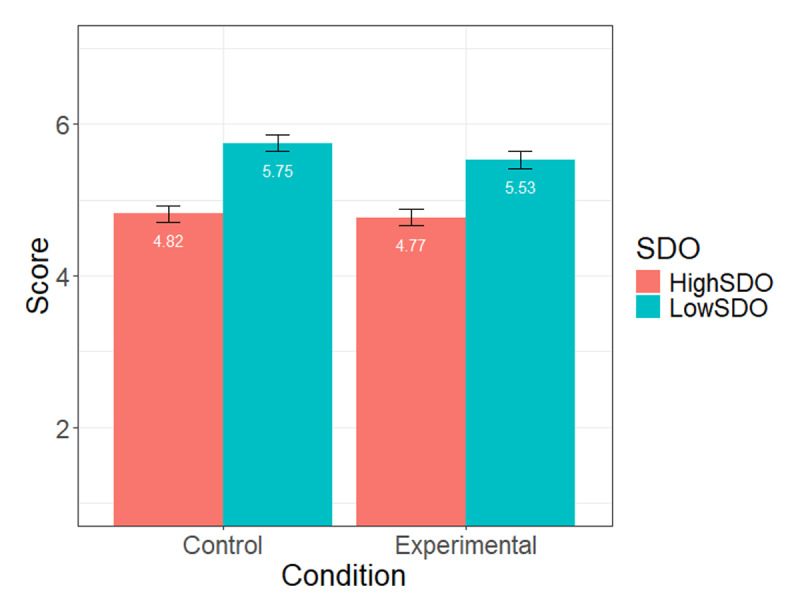
Mean level of Inclusion of people with language difficulties at high (+1SD) and low (–1 SD) levels of SDO, across conditions (Study 1).

First, we examined whether the manipulation check item was affected by participants’ condition, that is, whether participants’ exposure to the gender-fair language (experimental) condition led them to feel concerned by the use of the gender-fair language. This was not the case, *b* = –0.10 (SE = 0.19), *t* = –0.50, *p* = .62. We also tested whether this effect was only observed among high-SDO participants (i.e., if there was a condition × SDO interaction) – and it was not the case, *b* = 0.03 (SE = 0.17), *t* = 0.19, *p* = .85. Because a non-significant manipulation check does not necessarily mean that the experimental induction failed, we proceeded to test our hypotheses. We regressed inclusion of PDLs on SDO, experimental condition, and their interaction. Detailed statistics are displayed in Table 2. We found a negative main effect of SDO, *p* < .001, but no condition effect, *p* = .22, and no SDO × condition interaction, *p* = .43 (see Figure 1 for means and standard errors at each condition level and SDO level)^2^,^3^.

**Table 2:** Regressing dependent variables on experimental manipulation, SDO, and their interaction.


PREDICTOR	*b*	95% CI	*t*	*R^2^*

**Inclusion of people with language difficulties**				

(Intercept)	5.22**	[5.11, 5.32]		

SDO	–0.38**	[–0.48, –0.28]	–7.59	

Condition	–0.14	[–0.36, 0.08]	–1.24	

SDO × condition	0.08	[–0.12, 0.28]	0.79	

				.13**

**Support for a person with language difficulties**				

(Intercept)	3.52**	[3.34, 3.69]		

SDO	–0.11	[–0.27, 0.05]	–1.36	

Condition	–0.63**	[–0.98, –0.28]	–3.51	

SDO × condition	–0.22	[–0.54, 0.10]	–1.35	

				.041**


* *p* < .05, ** *p* < .01.

**Figure 2:**
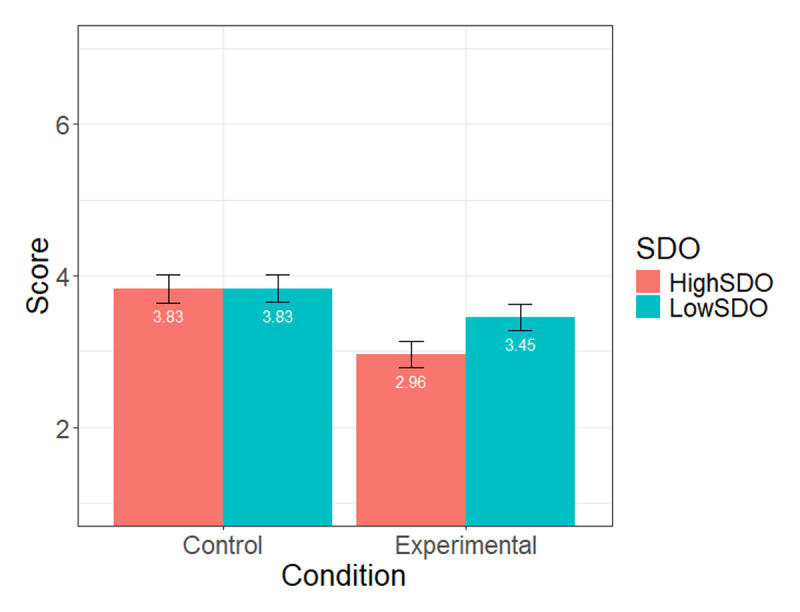
Mean level of support for a person with language difficulties at high (+1SD) and low (–1 SD) levels of SDO, across conditions (Study 1).

We then tested the same model to predict support for the PDL complaining about the current use of French (see Table 2 for detailed statistics). We found a main effect of the condition, *p* < .001, such that participants who read the vignette about gender-fair language showed more support for the person. However, we found no SDO effect, *p* = .17, nor SDO × condition interaction, *p* = .18 (see Figure 2 for means and standard errors at each condition level and SDO level).

### Discussion

In Study 1, we sought to induce a sense of threat by presenting experimental participants with a vignette discussing the increasing use of gender language in French-speaking countries – and the ensuing debates and concerns. Unfortunately, the vignette did not appear to elicit a significant level of concern among participants, even among those with high levels of SDO.

In this study, we tested our hypotheses using two dependent variables: inclusion of PDLs, but also support for the author of a fake Facebook comment complaining about the ‘current use of French’. In both cases, we failed to observe the expected interaction effects. Hence, we did not find evidence of ideological malleability in this study. We found a condition effect on the support for the PDL person, such that all participants reported higher levels of support for the person with language difficulties in the experimental condition. In other words, making gender-fair language salient increased support for the PDL complaining about the complexity of the French language. Finally, SDO was a negative predictor of the inclusion of PDLs. SDO was also negatively related to the support for the PDL complaining about French being inaccessible.

## Study 2

In Study 1, we manipulated threat associated with gender-fair language and expected high-SDO participants to mobilize support and inclusion of PDLs to reject gender-fair language. In this study, we failed to find support for our SDO × manipulation interaction hypothesis. A potential explanation for these non-significant findings lies in the fact that this study lacked contextual cues indicating that support for PDLs could be mobilized to oppose gender-fair language. Indeed, the core idea of malleable ideologies (and of the justification suppression model of prejudice) is that egalitarian values can be instrumentalized as justifications for one’s prejudice (Crandall & Eshelman, 2003; Knowles et al., 2009). Hence, malleable ideologies patterns should be more likely to emerge in a context that calls for the justification of one’s prejudice.

In Study 2, the interests of PDLs were explicitly presented as conflicting with the advancement of gender-fair language. Doing so, we created a context in which high-SDO individuals were offered a socially acceptable justification for their rejection of gender-fair language on a silver platter. We presented participants with a Twitter (now X) status expressing a refusal of special accommodations for dyslexics. In the experimental condition, this Twitter status was written in gender-fair language by a gender-fair language advocate, whereas in the control condition, it was written in conventional language by a ‘defender of the French language’. We also then presented them with a comment written by a dyslexic person reacting to the status. We examined their agreement with the status, support for the dyslexic person reacting to it, and inclusion of PDLs in general. The study was preregistered. Preregistration, materials, data and R script are available on the Open Science Framework website: https://osf.io/9za84/?view_only=7bf99ceb1451482f925eaa6dd834c332.

### Hypotheses

We hypothesized that SDO would be positively associated with agreement with the person expressing that ‘dyslexics should not be given special treatment’ (H1) and negatively with support of the dyslexic person (H2). To capture ideological malleability, we expected a SDO × condition interaction for agreement with the person expressing that dyslexics should not benefit from special treatment (H3). Specifically, we expected high-SDO participants to report less agreement with the Twitter status stating that dyslexics should not be given special treatment, when its author is described as a gender-fair language defender (i.e., experimental condition), compared to a condition in which the author is described as a French language defender (i.e., control condition). Similarly, we hypothesized a SDO × condition interaction in the model predicting support for the dyslexic person (H4). We expected high-SDO participants to report more support for the dyslexic person’s response to the Twitter status, when the author of the status was described as a defender of the gender-fair language. Conversely, we expected that low-SDO participants would show an equal agreement with person A expressing that dyslexics shouldn’t be given special treatment across both conditions. We also expected that they would express equal support of the dyslexic person across both conditions. Finally, we tested the SDO × condition interaction when predicting inclusion for PDLs – just like in Study 1. This hypothesis was exploratory.

### Methods

#### Participants

To detect a small minimum effect size (*r* = .10) with a statistical power of 80%, we needed 800 participants. We anticipated a 10% dropout rate, and therefore planned on recruiting at least 880 participants. We collected French participants on Prolific (n = 1069). After removing participants who failed attention checks, our sample comprised 788 participants (404 women, 375 men, 9 who identified to another gender, *M_age_* = 41.28, *SD_age_* = 118.28). A sensitivity analysis determined that 788 participants would still allow 80% power to detect an effect of *r* = .10. In terms of education, 13.8% had no diploma, a primary or secondary school diploma, 31.6% had completed one to three years of higher education, 54.6% had completed 4 years of higher education or more.

#### Procedure

After giving their informed consent, participants responded to the demographic questions and social dominance orientation scale. Participants were told that the questionnaire was about how people express themselves on social media and how their words are perceived. They then learned that they would be asked to react to two Twitter (now X) posts. Participants were randomly assigned in one of two conditions by showing them a fake Twitter status of a French-speaking man. In the control condition, the Twitter profile read ‘School teacher. Fighting for the defence of the French language’. In the experimental condition, the Twitter profile read ‘School teacher. Fighting for the defence of gender-fair language’. The account published a status stating that ‘It is impossible to grant special treatment for dyslexics. In the workplace, they must adapt to their boss’ requests’ (English translation). The sentence was the same in both conditions, however, in the experimental condition, it was written in gender-fair language, using the ‘*point médian*’ and the gender-neutral pronoun ‘*ielles*’ (‘Il est impossible d’offrir des conditions spéciales pour les dyslexiques. Au travail, ielles doivent s’adapter aux demandes de leur patron·nes comme les autres salarié·es’). In the control condition, the sentence was written in conventional French. Participants then responded to two attention checks and indicated their agreement with the person expressing that dyslexics should not be given special treatment. Next, we presented participants with a fake comment, supposedly written by a dyslexic reacting to the status. The comment read: ‘I’m dyslexic and I don’t agree. You should be supporting people who have difficulties with language instead of pushing us down’. Participants indicated their support of the dyslexic person and finally, their inclusion of PDLs in general. Finally, they were thanked and debriefed.

#### Measures

**Social dominance orientation (*α* = .88)**. Participants responded to the same scale as the one used in Study 1.

**Attention checks.** To ensure that participants read the material, we asked participants two attention check questions: ‘What is the job of this person?’ (correct answer: ‘school teacher’) and ‘This person seems to be committed. To which cause?’ (correct answer: ‘to the defence of gender-fair language’ or ‘to the defence of French’ depending on the condition). These questions appeared just below the fake Twitter account, so that participants were able to check the image to respond.

**Agreement with the teacher expressing that dyslexic people should not be given special treatment**. After reading the fake Twitter account and status, participants were asked ‘to what extent do you agree with this person’s position on the inclusion of dyslexic people?’ and responded on a scale ranging from 1 (not at all) to 7 (completely).

**Support of the dyslexic person**. Participants indicated their support with the dyslexic person (person B) on a scale from 1 (I do not support this person at all in their comment) to 7 (I fully support this person at all in their comment).

**Inclusion of PDLs (*α* = .86)**. The scale was the same as in Study 1, except for two items that were reversed (e.g., society is doing *far too much* to include people with language difficulties).

### Results

Table 3 presents a correlation matrix. Replicating findings from Study 1, and in line with our hypotheses, SDO was negatively associated with inclusion of PDLs, *r* = –.57, *p* < .001, positively related with agreement with the status stating that dyslexic people should not benefit from special treatment, *r* = .36, *p* < .001, and negatively related to support for the dyslexic person, *r* = –.39, *p* < .001.

**Table 3:** Means, standard deviations, and correlations with confidence intervals from Study 2.


VARIABLE	*M*	*SD*	1	2	3	4	5	6	7

1. Gender (–0.5 = man, +0.5 = woman)	*na*	*na*							

2. Age	41.28	18.28	.02						

			[–.05, .09]						

3. Education level	8.23	2.18	.00	–.12***					

			[–.07, .07]	[–.19, –.05]					

4. Political orientation	3.24	1.57	–.17***	.08*	–.04				

			[–.24, –.10]	[.01, .15]	[–.11, .03]				

5. SDO	2.22	1.10	–.23***	–.01	.01	.63***			

			[–.30, –.16]	[–.08, .06]	[–.06, .08]	[.58, .67]			

6. Agreement with person A (non-inclusion of dyslexics)	2.70	1.67	–.13***	.08*	.00	.33***	.36***		

			[–.20, –.06]	[.01, .15]	[–.07, .07]	[.27, .40]	[.30, .42]		

7. Support dyslexic person	5.91	1.34	.16***	–.01	–.03	–.33***	–.39***	–.54***	

			[.09, .23]	[–.08, .06]	[–.10, .04]	[–.39, –.27]	[–.45, –.33]	[–.58, –.49]	

8. Inclusion of PDLs	5.38	1.06	.25***	–.07*	–.03	–.51***	–.57***	–.53***	.60***

			[.19, .32]	[–.14, –.00]	[–.10, .04]	[–.56, –.45]	[–.62, –.53]	[–.58, –.48]	[.55, .64]


*Note. M* and *SD* are used to represent mean and standard deviation, respectively. Values in square brackets indicate the 95% confidence interval for each correlation. * indicates *p* < .05. ** indicates *p* < .01. *** indicates *p* < .001.

**Figure 3:**
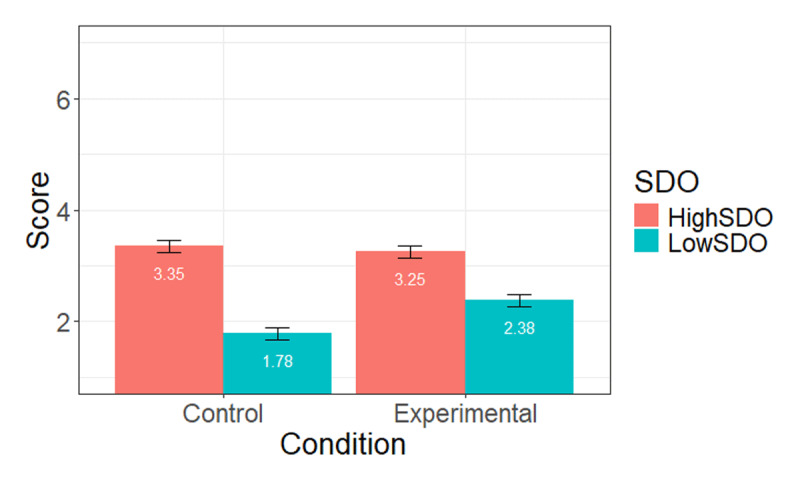
Mean level of agreement with the person stating that dyslexic should not be given special treatment, at high (+1SD) and low (–1 SD) levels of SDO, across conditions (Study 2).

We then examined our hypotheses regarding agreement with the person stating that dyslexic should not be given special treatment. The model including SDO, condition, and their interaction as independent variables (see Table 4 for detailed statistics). We observed a positive effect of SDO, *p* < .001. As expected, high-SDO participants agreed with the fact that dyslexics should not be given special treatment. We also found an unexpected positive effect of the condition, *p* = .02, such that participants expressed higher agreement with the status when its author was presented as a gender-fair language defender, and wrote in gender-fair language (i.e., experimental condition). Looking at the interaction between SDO and condition, we found a significant effect, *p* = .001. However, when examining simple slopes at –1 and +1SD, we found that high-SDO participants did not change their agreement across conditions, b = –0.10 (SE = 0.16), *t(784)* = –0.65, *p* = .52 (*M_Experimental_* = 3.25, *SE* = 0.11, *M_Control_* = 3.35, *SE* = 0.11), whereas low-SDO participants did, *b* = 0.60, *t*(784) = 3.88, *p* < .001 (*M_Experimental_* = 2.38, *SE* = 0.11, *M_Control_* = 1.78, *SE* = 0.11). Specifically, low-SDO reported more agreement, or less disagreement, with the twitter status refusing special treatment for dyslexics, when the status what written by a defender of gender-fair language, compared to a defender of the French language. Figure 3 shows the interaction pattern.

**Table 4:** Regressing dependent variables on experimental manipulation, SDO, and their interaction.


PREDICTOR	*b*	95% CI	*t*	*R^2^*

**Agreement with the anti-PDL adjustments statement**				

(Intercept)	2.69**	[2.58, 2.80]		

SDO	0.56**	[0.46, 0.65]	11.05	

condition	0.25*	[0.04, 0.47]	2.29	

SDO × condition	–0.32**	[–0.52, –0.12]	–3.20	

				.15**

**Support for the dyslexic person**				

(Intercept)	5.92**	[5.83, 6.00]		

SDO	–0.48**	[–0.56, –0.40]	–12.11	

condition	–0.10	[–0.27, 0.07]	–1.15	

SDO × condition	0.19*	[0.04, 0.35]	2.43	

				.16**


* *p* < .05, ** *p* < .01.

**Figure 4:**
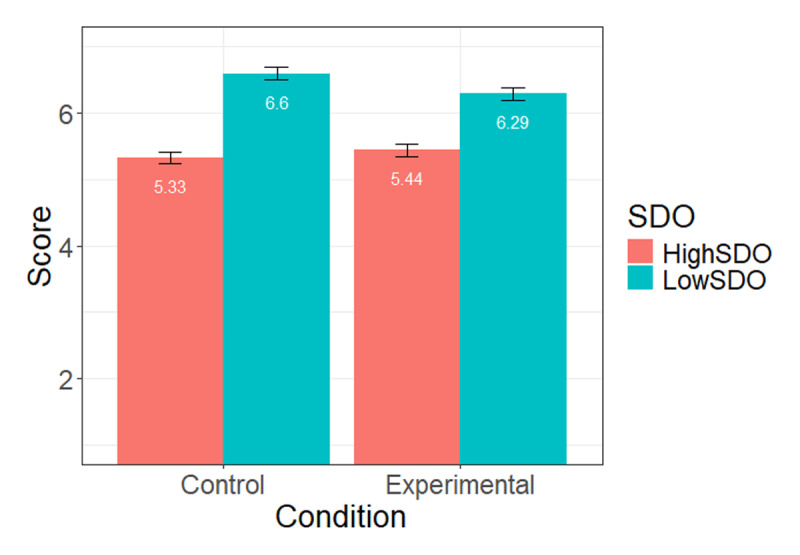
Mean level of support for the PDL complaining about the statement at high (+1SD) and low (–1 SD) levels of SDO, across conditions (Study 2).

In the model comprising participant’s SDO score and condition to predict support for the dyslexic person commenting the status, we found a main negative effect of SDO, *p* < .001, and no effect of the experimental condition, *p* = .25. We again found a SDO × condition interaction, *p* = .02 (see Table 4 for detailed statistics). Looking at simple slopes, the condition again failed to reach significance among high-SDO participants, *b* = 0.11 (*SE* = 0.12), *t*(784) = 0.91, *p* = .36 (*M_Experimental_* = 5.44, *SE* = 0.09, *M_Control_* = 5.33, *SE* = 0.09), but was significantly negative among low SDO, *b* = –0.31 (*SE* = 0.12), *t* = –2.53, *p* = .01 (*M_Experimental_* = 6.29, *SE* = 0.09, *M_Control_* = 6.60, *SE* = 0.09, see Figure 4). Thus, low-SDO participants showed less support for the dyslexic person in the experimental gender-fair language condition than they did in the control condition.

**Figure 5:**
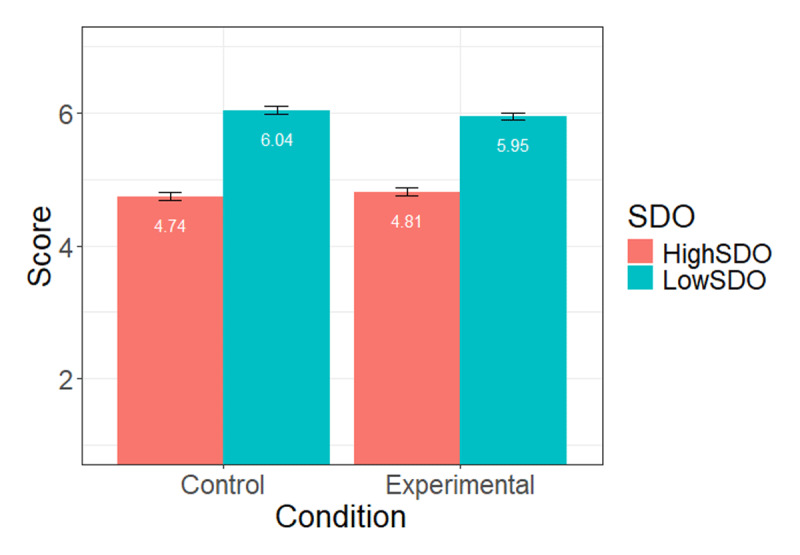
Mean level of inclusion of PDLs at high (+1SD) and low (–1 SD) levels of SDO, across conditions (Study 2).

Finally, we ran the same model with inclusion of PDLs as dependent variable. We found the expected negative effect of SDO, *b* = –0.56, *t* = –19.69, *p* < .001, but no effect of condition, *p* = .92 and no SDO × condition interaction, *p* = .17 (see Figure 5).

### Discussion

In Study 2, we presented participants with a manipulation in which the justification potential of the defense of dyslexics was more salient than in Study 1. Just like in Study 1, SDO was negatively associated with support for the dyslexic person and the inclusion of PDLs in general. Even though we found a significant interaction between SDO and the experimental manipulation, the shape of the interaction was not indicative of ideological malleability among high SDO participants. Instead, we found unexpected interaction patterns whereby low-SDO individuals shifted their agreement and support across conditions: When the twitter status was written by a defender of gender-fair language, they reported more agreement (or rather, less disagreement) with the status and showed less support for the dyslexic person challenging the status. High SDO participants reported more agreement with the status and lower support for the dyslexic person than low SDO participants but were unaffected by the experimental manipulation.

## General discussion

In light of the growing visibility of gender-fair language in France, many (far) right wing outlets and political figures objected that the enforcement of such language would constitute an obstacle for people who experience difficulties with the French language (PDLs). Since antiegalitarians tend not to be concerned about the inclusion of disadvantaged groups, we sought to examine whether high-SDO individuals would report an increased support for and inclusion of PDLs when confronted with the issue of gender-fair language. This hypothesis maps onto works on ideology malleability (Knowles et al., 2009) and the justification-suppression model of prejudice (Crandall & Eshelman, 2003).

Building on the theoretical rationale of Knowles and colleagues (2009) and on the methodology of subsequent works (Guimond et al., 2013), we attempted to experimentally manipulate gender-fair language threat either directly (in Study 1, with a vignette discussing the progress of gender-fair language in society) or, adopting a slightly different approach in Study 2, we had participants read and react to an anti-PDLs Twitter post that was shared by a gender-fair language advocate (vs. a French language advocate).

In both studies, we did not find evidence of ideological malleability—operationalized as an interaction between SDO and experimental manipulations in the prediction of support or inclusion of PDLs. Against our hypotheses, high SDO participants were not more likely to support people with language difficulties when the issue of gender-fair language was made salient, compared to a control condition. Can we conclude from these results that individuals do not weaponize PDLs to reject gender-fair language? We argue that several factors prevent us from making such a definitive claim. First, the discourse among certain political figures—whose political orientations and shifting interests in issues related to people with language difficulties are reflected in their policy positions—suggests that the phenomenon of weaponizing PDLs is observable among politicians. Second, in line with this, we consistently observed a negative relationship between Social Dominance Orientation (SDO) and inclusion PDLs and gender-fair language, suggesting that high-SDO individuals (among which, far right politicians) are in general less likely to be concerned about the inclusion of PDLs.

There are many possible accounts for why we failed to corroborate our hypotheses. A first, obvious reason resides in the difficulty of experimentally manipulating the independent variable, even when using a scenario explicitly referring to the tension between the promotion of gender-fair language and the support for dyslexic people (Study 2). In Study 1, the experimental manipulation did not have the expected impact on the manipulation check. Even though non-significant manipulation checks do not necessarily mean failed experimental inductions (i.e., it may be due to an inadequate measure used as a manipulation check), this situation emphasizes the difficulty to manipulate threat perceptions pertaining to gender-fair language. It is worth noting that while some previous studies manipulated more straightforward perceptions of threat (e.g., a scenario about a person being fired for expressing racist views, White & Crandall, 2017), our manipulations were more indirect—especially in Study 3. Although in some studies, mere topic salience or self-categorization as a member of the privileged majority (e.g., Knowles et al., 2009; Morrison & Chung, 2011) may have been enough to elicit threat perceptions among high-SDO participants, this may not be the case when investigating gender-fair language. A more threatening manipulation (e.g., using a scenario about a person being fired for refusing to comply to gender-fair language company policies) may have yielded different results.

Another possible reason lies in the approach used to measure the malleability of ideology, and particularly the support for PDLs. Although various studies have highlighted ideological malleability using close-ended questions to measure ideology endorsement (e.g. Knowles et al., 2009), it is possible that closed-ended questions, and/or online studies, do not constitute the best opportunity for participants to show justification processes. On this front, open-ended questions may encourage participants to explain better their position, thus revealing how they mobilize egalitarian ideologies (see Van Oost et al., 2023 for this approach).

A third reason for the difficulty in capturing the weaponization of PDLs is that only a specific subgroup of antiegalitarian individuals—especially those who are more politically active and thus aware of this weaponization—may engage in this behavior. As a result, our focus on measuring antiegalitarianism and using specific experimental conditions may not have fully captured this phenomenon, which could be more pronounced within a smaller, more politically engaged group. Instead of ideology malleability, we observed that SDO was consistently associated with increased rejection of gender-fair language, and decreased inclusion of PDLs. Hence, while it is possible to find instances of antiegalitarians advocating for the inclusion of PDLs, antiegalitarians are—somewhat unsurprisingly—less supportive of these people. Although we failed to find evidence for ideological malleability in an experimental setting, these findings suggest that real life occurrences of far-right wingers expressing support PDLs against gender-fair language do not reflect a defense of a disadvantaged group as much as the rejection of gender-fair language. By way of contrast, the positive relationships consistently observed between support for gender-fair language and inclusion of PDLs mitigates the concern that the promotion of gender-fair language may overshadow the needs of people who experience difficulties with language. It is worth noting that in France, associations took publicly position against what they perceived as an opportunistic defense of disabled people by the political right (Réseau d’Études HandiFéministes, 2020).

Interestingly, Study 2 found an unexpected interaction between SDO and gender-fair language salience that certainly deserves our attention. The pattern of simple slopes was opposed to what we hypothesized: Whereas high-SDO were unaffected by the experimental manipulation, low-SDO reported an increased endorsement of an anti-PDLs status, and decreased support for a PDL that complained about it. This effect may not capture a malleable ideology effect as much as a form of ingroup bias, or a motivated reasoning driven by shared political values (Kahan, 2015): participants who supported gender-fair language were likely motivated to agree with the author of the Twitter status, because they share a political cause, resulting in less support for the PDL complaining about the author. This suggests that even though egalitarians are more supportive of members of marginalized groups, some contexts are susceptible to reduce their support – notably, when the interests of a stigmatized minority clash with a statement made by an ingroup member (i.e., a person who sympathize with a same cause, see Bliuc et al., 2007). A similar – yet stronger – tendency has been shown among Chinese antiauthoritarians, whose antiauthoritarian preferences (e.g., rejection of punitive justice) either reversed or disappeared in situations of resources loss (Johnson et al., 2022). It is however important to stress that the agreement with the status hostile to accommodation for PDLs was on average higher among high-SDO than among low SDO participants.

## Limitations and conclusion

The main limitation relies in the absence of evidence indicating that we successfully manipulated threat perceptions among high-SDO participants, in spite of our efforts to rely on various approaches (e.g., mere topic salience following the work of Morrison & Chung, 2011; reading of vignette emphasizing increase of hierarchy-challenging practices, following the work of Knowles et al., 2009; or reading of a controversial stance associated with the ideology at hand, following the work of White & Crandall, 2017).

Another limitation lies in our decision not to test moderation with right-wing authoritarianism (RWA). The literature highlights various moderators that explain how ideologies and values are leveraged by less egalitarian or conservative individuals. For instance, malleable ideologies are often studied in connection with individuals high in SDO (Chow & Knowles, 2016; Knowles et al., 2009; Unzueta et al., 2012; Roebroeck & Guimond, 2018). Similarly, prejudice has been used as a moderator to explore how ideologies are mobilized in interracial contexts (e.g., White & Crandall, 2017; White et al., 2021). It is reasonable to suspect that right-wing authoritarians may employ a similar strategy, particularly when issues at stake are perceived as threatening cultural continuity (Duckitt & Sibley, 2007)—as is the case for gender-fair language. In our study, building on Knowles et al.’s, we focused on moderation via SDO, given its relevance to resisting gender-fair language as a means of maintaining social hierarchies. However, future research could benefit from examining moderation through RWA as well. Of note, our findings also revealed in Study 1, political orientation was more strongly related to two of the three dependent variables than was SDO (inclusion of PDLs, and attitudes towards gender-fair language). This suggests that political orientation may be a broader and potentially more predictive construct in this context, as it likely captures both beliefs about acceptance towards inequality (proximal to SDO) and resistance to social change (proximal to RWA; Jost et al., 2009). Thus, while we relied on SDO, RWA, or political orientation (which captures both ideological beliefs regarding inequality and social change) would perhaps have yielded different results. Although this is an important limitation, we believe these studies are informative as to which approach should be privileged (or avoided) when it comes to induce perception of intergroup threat in the context of politicized issues such as gender-fair language. Across two experimental studies, we did not find evidence that high-SDO were more likely to support people who have difficulties with language when confronted to the topic of gender-fair language. Thus, we failed to conceptually replicate ideological malleability in the context of language fair language in France. Instead, SDO was consistently negatively associated with support for PDLs and support for gender-fair language. By contrast, support for gender-fair language was positively associated with concern for PDLs. Finally, in Study 2, we found that low-SDO participants were less likely to support PDLs when hostility to adapt language to the special needs of PDLs came from a gender-fair language advocate. High-SDO participants, however, were overall less supportive of PDLs than low SDO, regardless of the experimental condition.

To our knowledge, this is the first study to investigate the relationships between SDO, attitudes towards people with language disabilities, and attitudes towards gender-fair language. Moreover, by highlighting some of the difficulties associated with the manipulation of intergroup threat in the context of malleable ideologies, our research is clearly informative for future attempts to conduct similar studies.

## Additional File

The additional file for this article can be found as follows:

10.5334/pb.1342.s1 Supplemental Material. Appendix 1–3.

## Footnotes

In total, three studies were conducted. In a first study, we failed to find the expected ideological malleability effect – like in the reported studies. Because of the difficulty to interpret these null results, and following advice from the editor, we moved the Study to supplemental materials (Study preregistration, materials, data files, Rscripts as well as results, are available on OSF: https://osf.io/rw7cg/).

We also proceeded to test whether using the subdimensions of SDO (SDO-D, and SDO-E) as moderators would yield different results. Throughout our three studies, we find that the results remain largely similar: in instances where the interaction of SDO-condition was unsignificant, it remains unsignificant when using both SDO-E and SDO-D. These analyses can be found in the R script on the OSF.

We ran additional set of analyses, testing whether the addition of demographic variables (i.e., gender, age and political orientation, separately) would yield different results on our interaction of interest. In our two studies, the addition of these variables does not change the results. Specifically, in instances where the interaction of SDO-condition was unsignificant, it remains unsignificant when adding a control variable. It does not yield different results with regard to the unexpected interaction patterns found in Study 2 either. These analyses can be found in the R scripts on OSF.
