# Supplementary material for: Do Anti-Egalitarians Report Increased Support for People with Language Difficulties when Exposed to Gender-Fair Language?
Source: Psychol Belg. 2025 May 22;65(1):132–45. doi: 10.5334/pb.1342 (PMC12101109; doi:10.5334/pb.1342)
Supplement: Supplemental Material. — Appendix 1–3. [file pb-65-1-1342-s1.pdf]

Supplemental material

Appendix 1. Vignette text from the experimental condition, Study 2

Depuis quelques années, l'écriture inclusive, et plus généralement, le langage inclusif sont de plus en plus présents dans notre quotidien. Quel est le but derrière ces nouvelles pratiques ? Démasculiniser le langage, qui serait la marque de la domination masculine et qui perpétuerait le sexisme et le patriarcat.

Si vous parlez d'une femme, ne dites plus « chef », mais « cheffe », ne parlez plus des « droits de l'Homme » mais des « droits humains » ou « droit de la personne ». On écrira « étudiant·e·s ». A l'oral, on dira « iel » (contraction de il et elle, notamment pour parler des non-binaires), « celleux » (celles et ceux), et « agriculteurices ».

La pratique gagne du terrain a une vitesse impressionnante. Chez nos voisin·e·s belges francophones, elle va être préconisée à l'écrit comme à l'oral au niveau de toutes les institutions subventionnées (médias, écoles, etc.). Au Canada, le gouvernement fédéral donne des directives en ce sens.

En France aussi, cette manière d'écrire s'impose de plus en plus dans les médias et les entreprises. Le Haut conseil à l'égalité entre les hommes et les femmes défend l'écriture inclusive, pour cesser « d'invisibiliser les femmes ». Il recommande aussi d'enlever l'expression « mademoiselle », jugée sexiste. Certain·e·s s'inquiètent que ces nouveaux usages détruisent la langue française.

Appendix 2. Vignette text from the control condition, Study 2

Les albatros sont des oiseaux de mers. Leur nom « scientifique » correspond à la famille des diomédéidés. La particularité de cette famille est leur grande envergure. Ils sont parmi les oiseaux volants ayant la plus large envergure, avec le grand albatros et son une envergure pouvant atteindre 3,50 mètres. Cette envergure rend leur envol difficile (les albatros doivent courir pour parvenir à décoller) mais leur permet de planer sans fournir d'effort. Ils sont très efficaces dans l'air, planant et volant sur des durées et des distances très longues. Les albatros adultes mesurent en moyenne autour d'un mètre (71 à 135 centimètres) et ont un pelage de diverses teintes de gris et de blanc, proche de celui des mouettes. Les jeunes albatros mettent des années à acquérir leur plumage adulte.

Leur régime est varié et constitué de zooplancton, poissons, céphalopodes et crustacés, parfois de charognes aussi, bien que le régime dépende d'une espèce à l'autre et d'une population à l'autre. Ils trouvent notamment leur nourriture à la surface de l'eau, mais peuvent aussi plonger à faible profondeur, certaines espèces plongeant jusqu'à 5 mètres sous l'eau pour attraper leur proie.

Pour pondre, les albatros effectuent leur reproduction en colonies, le plus souvent sur des îles isolées. Le poussin est couvé, nourri, et mettra entre 140 et 280 jours pour pouvoir voler.

Appendix 3: Supplemental Study

In this Study, we wanted to examine whether merely making the issue of gender-fair language salient would encourage high-SDO participants to express higher support for PDLs. In order to test this hypothesis, we used a 2 (salience of gender-fair language vs. control condition) × 2 (high vs. low SDO) design with both variables varying between participants. The manipulation of salience consisted in changing the presentation order of scales (see Guimond et al., 2013; Morrison et al., 2011), such that participants in the salience condition were presented with the gender-fair language scale before the supports for PDLs, while participants in the control condition saw scales in the reverse order. The study was preregistered. Preregistration, materials, data files and analyses scripts are available on the Open Science Framework: https://osf.io/rw7cg/

Hypotheses

For this Study, we hypothesized that the defence of PDLs would be negatively correlated with SDO (H1). We also hypothesized a positive association between the defense of PDLs and the favorability to gender-fair language (H2), as both attitudes are expected to be motivated by egalitarianism. Finally, we expected an experimental condition × SDO interaction in the prediction of support for PDLs (H3). Specifically, we expected that high-SDO participants would have a more positive attitude towards the inclusion of PDLs when they have been exposed to the issue of gender-

fair language (i.e., experimental condition) compared to a condition where they do not have been exposed to the issue of gender-fair language (i.e., control condition). Conversely, we expected low-SDO participants to show equally positive attitudes towards inclusion of PDLs in both conditions. The study was preregistered.

Method

Participants

Detecting a small minimum effect size of *r* = 0.10 (Funder & Ozer, 2019) with a power of 80% requires 800 participants. To improve data collection efficiency while controlling type 1 error risk, we made use of sequential analyses (Lakens, 2014). Thus, we planned to collect a maximum of 800, with an interim analysis planned halfway through sample completion (n = 400) to have an equal number of observations between each look (Lakens, 2014). As recommended in the Pocock boundary method, the interim analyses were conducted with the adjusted p-value threshold of 0.0294.

We collected our data using Foule Factory. Due to issues with the platform, we only managed to recruit 343 participants. Because we did not find the expected effect at our intermediate sample, we terminated the data collection.

We excluded participants who did not complete the two attention checks, reaching a total of 329 participants (176 women, 153 men, and 0 did not wish to indicate their gender, M_{age}= 43.28, SD_{age}= 12.6). In terms of education, 28% had no diploma, a primary or secondary school diploma, 46,5% had completed one to three years of higher education, 25.5% had completed 4 years of higher education or more.

Procedure

Participation took place online. After giving their informed consent, participants responded to a series of scales whose order varied depending on the experimental condition, as inspired by Guimond et al., 2013 and Morrison et al. (2011).In the experimental condition, after reporting their demographics, participants completed the gender-fair language scales before completing the inclusion of PDLs and SDO scales. In the control condition, the order of the inclusion of PDLs and gender-fair

scale was reversed, such that participants were exposed to the issue of gender-fair language before reporting their support for the inclusion of PDLs. At the end, all participants were thanked and debriefed.

To clarify, in this Study, we aimed to operationalize the concept of threat through a minimalist salience manipulation. Specifically, we hypothesized that simply drawing participants' attention to the topic of gender-fair language would be sufficient to elicit feelings of threat, given the sociopolitical context in France, where this issue has been the subject of intense debate and controversy. For instance, gender-fair language has faced significant opposition, including its prohibition in educational settings. This highly charged environment likely amplifies the sensitivity to even minimal reminders of the concept, making salience alone a plausible means of triggering perceived threat.

Measures

All variables were measured using a seven-point Likert scale ranging from completely disagree (1) to completely agree (7), with 4 indicating “neither agree nor disagree”.

Gender-fair language attitudes (α = .91). Participants answered a series of items about gender-fair language. For the first three questions, we created short scales by selecting items from the ’Inventory of Attitudes Towards Sexist/non-Sexist Language-General’ (Parks & Roberton, 2000) which consists in three sections, and adapted them to the French-speaking context. Examples of items are ‘When teachers teach history, they should not talk about ‘prehistoric man’ but about ‘prehistoric human beings’ or ‘prehistoric men and women’’, (1=strongly disagree, 7=strongly agree). Moreover, to enhance the sense of threat posed by gender-fair language, we created a 5-item scale pertaining to the ‘point médian’, a gender-fair language practice collapsing the masculine and feminine forms (e.g., “étudiant·e·s” to designate students that identify as men and women) that raised significant public attention (e.g., ‘When a secretariat sends an email to a mixed student group, it is normal for the email to begin with [Cher·e·s étudiant·e·s]’). Because the principal component analysis indicated unidimensionality, we computed a single mean score for all gender-fair language items.

Inclusion of persons who have difficulties with language (PDLs, *α* = .88). Participants responded to the same scale as in Study 1.

Social dominance orientation (*α* = .88). Participants responded to the same scale as in Study 1.

Results

Table 1 presents a correlation matrix of variable of interest. In line with H1 and H2, inclusion of PDLs was negatively associated with social dominance orientation (r=-.32, p < .001) and positively associated with positive attitudes towards gender-fair language (r =.35, p < .001).

Table 1 (Supplemental)

Means, standard deviations, and correlations with confidence intervals (Supplemental Study)

Variable	*M*	*SD*	1	2	3	4	5	6
1. Gender (-0.5 = man, +0.5 = woman)	*na*	*na*						
2. Age	43.28	12.63	.04<br>[-.07, .15]					
3. Education level	6.92	2.33	-.10<br>[-.21, .01]	-.20**<br>[-.30, -.09]				
4. political orientation	3.90	1.43	-.15**<br>[-.26, -.05]	.03<br>[-.07, .14]	-.12*<br>[-.22, -.01]			
5. SDO	2.44	1.10	-.13*<br>[-.23, -.02]	-.02<br>[-.13, .09]	-.03<br>[-.14, .07]	.35**<br>[.25, .44]		
6. attitudes towards gender-fair language	3.61	1.19	.12*<br>[.02, .23]	-.12*<br>[-.22, -.01]	-.04<br>[-.15, .07]	-.37**<br>[-.46, -.27]	-.28**<br>[-.38, -.18]	
7. Attitudes towards PDLs	5.36	1.01	.13*<br>[.03, .24]	.06<br>[-.05, .16]	-.10<br>[-.21, .01]	-.25**<br>[-.35, -.15]	-.32**<br>[-.42, -.22]	.35**<br>[.25, .44]

Note. M and *SD* are used to represent mean and standard deviation, respectively. Values in square brackets indicate the 95% confidence interval for each correlation. * indicates $p < .05$. ** indicates $p < .01$ *** indicates $p < .001$.

Before testing our hypothesis, we ensured that our moderator, social dominance orientation, was unaffected by the experimental manipulation – which was the case, $b = -0.02$ (SE = 0.06), $t = -0.27$, $p = .78$. Then, to test H3, we regressed support for the inclusion of PDLs inclusion on SDO (centered), experimental condition (coded -1 for control condition, 1 for experimental condition), and the interaction between these variables. Results with detailed statistics are displayed in Table 2. Social dominance orientation had a negative effect on PDLs inclusion, $p < .001$. Condition had no significant effect on support for PDLs, $p = .21$, and we did not find the expected interaction between SDO and condition, $p = .79$. Figure 1 shows the mean levels at each condition level and SDO level (+1SD vs. -1SD). The interaction did not emerge either in a model controlling for participants' score on Attitudes towards gender-fair language[1], or for gender, age or political orientation.

Table 2 (Supplemental)

Regressing attitudes towards the inclusion of people with language difficulties on experimental manipulation, SDO, and their interaction.

Predictor	*b*	95% CI	*t*(325)	R^2
(Intercept)	5.36**	[5.26, 5.47]		
SDO	-0.30**	[-0.39, -0.20]	-6.11	
Condition	0.07	[-0.04, 0.17]	1.26	
SDO × condition	0.01	[-0.08, 0.11]	0.26	
				.11**

* $p < .05$, ** $p < .01$.

Figure 1 (Supplemental)

Mean level of support for people with language difficulties at high (+1SD) and low (-1 SD) levels of SDO, across conditions (Supplemental Study)

[1] We regressed support for the inclusion of PDLs inclusion on SDO (centered), experimental condition, the interaction between these two variables, as well as attitudes towards gender-fair language (centered). In this model, we found again a negative effect of SDO, b=-0.22, $t(324) = -4.63$, $p < .001$, no effect from condition (p=.25), and again no SDO*condition interaction (p=.90). We found a positive effect of gender-fair language attitudes, b=0.24, $t(324) = 5.33$, $p < .001$. This analysis can be found in the R script, available on the OSF webpage.

Figure 1. Agreement with the inclusion of PDLs, depending on the condition and SDO level (Supplemental Study)

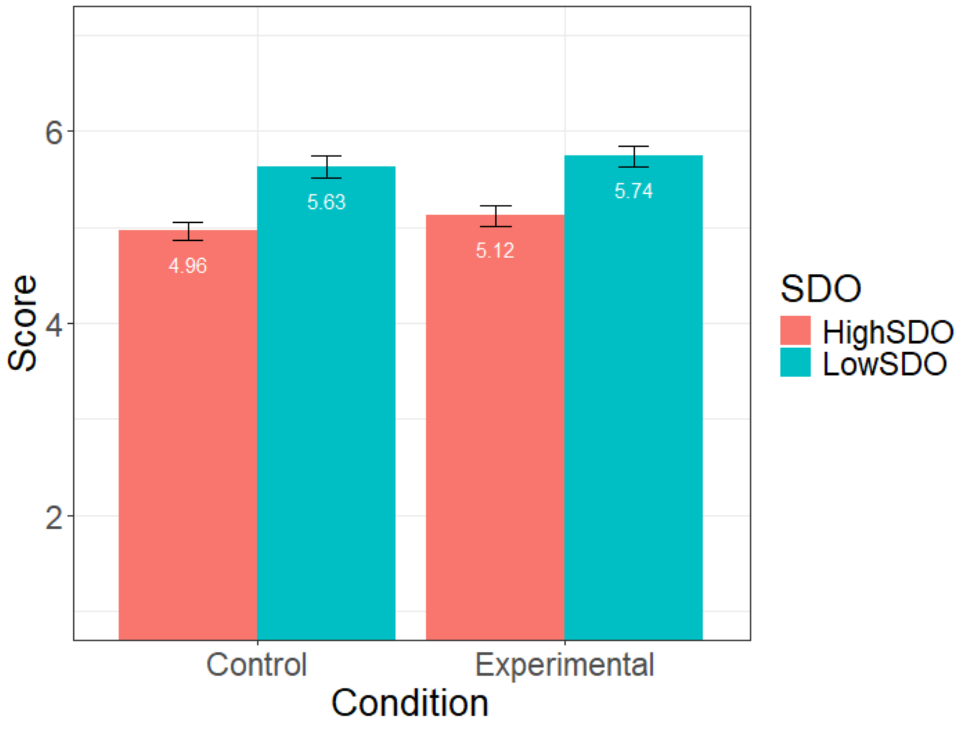

Discussion

In this first study, participants reported their attitudes towards gender-fair language, support for PDLs, and SDO. By manipulating the presentation order of the scales, we wanted to see whether making gender-fair language salient would shift high SDO participants' endorsement of PDLs inclusion. We did not observe the expected interaction. SDO was negatively correlated with support for PDLs, regardless of the condition. Hence, this first study failed to demonstrate an instrumentalization of the inclusion of PDLs by high SDO confronted to gender-fair language. However, the negative association between social dominance orientation and support for the inclusion of PDLs suggests that antiegalitarian tend not to be concerned by this group. Conversely, the positive association between support for gender-fair language and concern for the inclusion of PDLs suggest that gender-fair language supporters do not overlook the specific needs of PDLs. The absence of condition and interaction effect may mean that the manipulation failed to make gender-fair language sufficiently salient, or that mere topic salience did not induce a sense of threat among antiegalitarian participants. Another potential issue lies in participants' consistency striving in the experimental

condition: participants who first completed the gender-fair language scale may have been motivated to align their attitudes towards people with language difficulties with their attitudes towards gender-fair language. Since ideology malleability boils down to inducing inconsistency in participants’ responses (with anti-egalitarian participants reporting egalitarian answers), consistency striving may have played against the emergence of the hypothesized moderation.
